# Dental Treatment of Children

**Published:** 1889-04

**Authors:** Garrett Newkirk

**Affiliations:** Chicago.


ARTICLE II.

### DENTAL TREATMENT OF CHILDREN.

BY DR. GARRETT NEWKIRK, CHICAGO.

We often make a mistake in our estimate of character in placing children at too great a distance from their elders.

If men are "but children of larger growth" it follows that children are but little men and little women. They have in them the same elements of human nature, and exhibit fully as much variety of disposition. They have simply less development and experience, and are usually but not always more impulsive. They have not had the experience which enables some adults, not all, to exercise self control.

But on the whole, I have not been able to detect any great essential differences between children and their elders. The elder has had some additional mental growth and training. If the training has been good, it makes him the better patient; otherwise he may be worse, his mind filled with unreasonable notions, dreads and fears. I should prefer the child untrained to the adult ill-trained. I should prefer a patient with all his experience to get, to one whose experience has been unhappy and misleading.

At the outset we are quite apt, I think, to underrate the intelligence of children. They are often treated practically on the assumption that they know nothing, while the fact is they know a good deal. It is assumed that they are incapable of an understanding as to why things are done, but should simply submit out of deference to their elders and as a matter of course.

There is one thing children know and seem to know instinctively, and that is who like them and who don't. They know the sympathetic touch from the unsympathetic, and recognize their friends even among strangers.

If any dentist is affected with the mania of child hatred, if he looks on children generally as being merely in the way and better out, as far as his practice is concerned, he may as well leave them out. He will only arouse all their worse elements, and if they have any spirit it all will be manifested in well deserved antagonism.

An adult who has been trained to habits of cool calculation, may endure pain at the hand of one he dislikes, and who dislikes him for the sake of results, but children are not equal to such hypocrisy.

Any dentist who hopes to do a family practice is all wrong in permitting himself to cultivate such a disposition toward children. Children cannot help being children, with all the peculiarities incident to childhood.

As dentists, they are on our hands and we cannot evade the responsibility which devolves on us. It is the part of wisdom therefore, to recognize the facts and govern ourselves accordingly. We frequently hear it said: "I haven't the patience." Well, if you haven't, the quicker you set to work to acquire it the better. It is our *business* to have patience; we *must* have it. It is a confession of weakness to say we have it not. It is shameful to cultivate impatience.

Now, as to some points of difference in disposition. I have found that some children have considerable reasoning power at a very early age. One four years of age could not be induced to take castor oil, the taste of which he abhorred above all things, but when its action was explained, and the reasons for its use given in simple terms, he took it like a Major. This child endured several operations on his teeth when the uses of filling had been explained to him. He was very strong willed and often obstinate, but when he was made to understand that the operation proposed would be valuable to himself, his will became an ally instead of an antagonist.

*A reasonable child*, with a spirit and temper of his own, will consent to the endurance of pain if the consideration of a benefit to be derived is explained. With a child of this sort it is necessary to have an understanding beforehand with regard to what shall be done, the time required, etc. He will not submit without an agreement, and he will not agree without what seems to him good and sufficient reason. Especially is this true regarding first operations, before one has acquired the child's full confidence.

*Attention*. Sometimes it is difficult to get this long enough even with a reasonable child, to make him understand what you purpose doing. Here a dentist needs to be something of a teacher. Often the child, like the adult, is a victim of false education, and comes to the office with his head full of wrong ideas. His preconceived notions and his fears prevent the calm and logical action of his mind. No time can be better spent than in getting acquainted with such a child. Whatever is done, whether it requires one visit or three, his apprehensions must be sufficiently removed to permit of a reasonable talk and understanding, before any operation of importance is attempted.

*The curious child*—the child full of curiosity. I think he is in the majority, but there are some especially curious with reference to things mechanical. Such children are usually of a friendly disposition, and if greeted cordially, made to feel at home, are readily approachable by the way of their curiosity. A great many things in the office are objects of immediate interest to them; they ask many questions which should be answered as satisfactorily as possible. Such a child should be given a hand glass, that he may see his own teeth in the preliminary examination, and then during operations so that he may view the various steps taken and their final results. His interest is half the battle won. It is unfair, unwise and unkind to require such a child to just sit and submit to whatever we choose, without giving him anything to do, or to divert his attention. Under such circumstances the sitting becomes simply an unrelieved tension on his nerves. Many adults find such treatment hard enough, and who can blame the child whose nerves refuse to bear the strain ? As a principle of good common sense give the child somewhat to think about besides the one thing of simply enduring pain.

*The nervous child.* This type is well illustrated by the case of little Alice G., aged 7. She has been in the chair quite often, and I have filled several of her teeth, including one devitalized. She has never been hurt much. She is brave, but still has a nervous dread of every new operation proposed. It became necessary a few weeks since, to remove the almost rootless crown of a lower deciduous molar (her first bicuspids are coming very early). For fully five minutes she sat in the chair trying to get courage to let me apply the forceps. She would resolutely place her hands on the arm of the chair, steady her head in the rest, open her mouth; but just at the critical moment up the hands would go in spite of herself. Beads of perspiration stood on her forehead, and she would say, "I can't, Oh, indeed, I can't!" After several attempts with the same result, I said, "Well, we won't try any more now; we will wait till another day." I sat down by my desk, called her to me, and we had a quiet talk. I explained to her the conditions surrounding the tooth, its relations, to the new one coming, and that its removal would not be very painful. She said, I want it out, and I think I will be brave, but such an awful fear comes over me, I don't know why it is, but I tremble all over." Now, what sort of a man is he who could have any feeling other than tender sympathy for such a child as that?

Before she left the office she came to me and said, "Doctor, I believe I can have it out now, really," and she did. Her will had conquered; kind words, a little time to rest and to think had smoothed her unruly nerves, and she went through the operation beautifully.

*The proud child* is one whom we wish to treat with considerable deference and respect, as tho he or she were already quite a man or woman. It is best to assume with him that, of course, he is going through all right. He is apt to feel it would be a disgrace if some other child of his own age or younger should endure operations better than he; and so you may appeal strongly to the proud child by a casual mention of the operation you have done for Johnny D. or Mary G., and how quiet and brave they were. Without saying anything he will determine in his own mind not to be outdone by them.

There is also the child who may or may not be proud, but who particularly loves approbation. It pays to give him all the praise he deserves, and possibly a little more to take notice of his possessions and whatever pertaining to himself may be admired. Our appreciation of him and his is the best evidence, in his estimation, that we are worthy of confidence.

On the other hand we find children, as we do adults, who are not particularly intelligent or reasonable; not specially nervous or timid; not proud, and with very little, apparently, of approbativeness in their dispositions. Their ears are dull to argument, they don't care what others have had done, and they don't seem to care what anybody thinks of them; tho this is usually a blind; because to some extent, they do care. But they are *secretive*, and they are *obstinate*. They would appear to be just made up of a great big *I won't*.

In addition to or without this simple obstinacy of character another has a good deal of active combativeness. He has been born belligerent. His first cry was a bugle call to arms; his first hat carried a chip. "He smelleth the battle afar;" expects martial music wherever he goes, and especially with the dentist, whom he instinctively feels is a born adversary.

There are several ways of dealing with the obstinate or the combative child who is more frequently a boy than a girl. One way is to lose one's temper and general self-control. This means failure with a child of any age from six to sixty. One way with the combative child is not to oppose a combative spirit to his. If he seems determined not to let us do what may be necessary; say to him kindly, but firmly, "Very well, I cannot compel you to be a good boy. I am willing to do what I know will be for your good, but it is your interest, not mine, that is concerned; it really makes no difference to me, and it shall be just as you say." Throwing all the responsibility on himself takes him by surprise and turns his thoughts into a new channel. Refusing to have a combat with him throws him "off his base," so to speak. This sort of let-alone policy, especially with the older boys, will often have the effect of sobering them very quickly.

The obstinate child, as before marked, is usually secretive, and you can scarcely tell at times what he really thinks or intends to do. Often, however, he is at heart of a friendly disposition toward those who have once acquired his confidence. With such, especially the smaller ones, it is best to skirmish awhile, till one finds some place favorable for attack. Once penetrate the outer works of their resistence, get a little acquainted ; in other words, approach them on some ground of mutual interest, and they become as faithful friends as one could wish.

It is a doubtful experiment to attempt to control any child by sheer physical force. If a child submits, as he may do after a brief resistance, from a sheer sense of helplessness, it may be only with determination never to enter a dental office again. Unless the case is of extreme emergency, and as a last resort, the exercise of physical control a necessity, I should certainly prefer to let the child go indefinitely, waiting for his own consent to operations.

*Moods.* Children, like grown people, are subject to moods; they are not always the same. They are different on different days, according to bodily conditions and states of the weather. If conditions seem to be unfavorable; if the child is more than usually nervous; less self-controlled, it is better to let him go home with little or nothing accomplished beyond an examination. Especially is this true of first visits.

Now, I seem to hear objections to all this, that it takes too much time. It is true, primarily it seems a waste of time. We cannot always charge for it, but in the end it will pay, if not in money, certainly in moral influence. But, as to the time, we may avoid loss to some extent, by making short appointments. Appointments for children should always be short—seldom, if ever, more than half an hour, and often less.

I add a few general rules and considerations.

First. With all children we should be strictly truthful, not deceiving them ourselves, not permitting others to do so in our office. Confidence is everything, and cannot be obtained by deception.

Second. Next to truthfulness as to facts, genuine kindliness and friendliness; make them feel that we have their interests at heart.

Third. Treat them more like ladies and gentlemen than it is usual for people to do. Appeal to their sense of manliness and womanliness. Use gentle tones in speaking to them—this has often a wonderful influence, and not alone with children.

Fourth. Remember that *patience is first and last, and has no limit.*

Fifth. We should be exceedingly careful in our manipulations. All nature cries anathema on the man who will needlessly or carelessly torture a child. *It is imperative that we have delicate instruments kept in delicate order, and handle them delicately in the mouths of children.—Dental Review.*

